# P-2194. Prevalence of Hepatitis B and Hepatitis A in People with Chronic Active Hepatitis C in New Jersey

**DOI:** 10.1093/ofid/ofae631.2348

**Published:** 2025-01-29

**Authors:** Jihad Slim, Maria Akiki, Barbara Tempalski, Corey Rosmarin-DeStefano, Juan Torres, Kevin Leyden, Sheena Duprey, Emily Levaggi

**Affiliations:** Saint Michael’s Medical Center, Newark, NJ, USA, Newark, New Jersey; New York Medical College, Newark, New Jersey; NJCRI, Newark, New Jersey; North Jersey Community Research Initiative, Newark, New Jersey; NJCRI, Newark, New Jersey; NJCRI, Newark, New Jersey; North Jersey Community Research Initiative, Newark, New Jersey; NJCRI, Newark, New Jersey

## Abstract

**Background:**

Most current guidelines recommend hepatitis A (HAV) and hepatitis B (HBV) vaccination for people living with hepatitis C (HCV) and lacking immunity to those viruses. We were interested in studying the prevalence of immunity to those 2 viruses in patients with chronic active HCV infection (CAH C) in NJ, to evaluate the resources needed to vaccinate those who are not immune.

**Methods:**

We reviewed prospectively collected data in people with CAH C living in NJ, and retrieved their age, sex, race, ethnicity, HAV IgG, HBV surface antigen, HBV surface antibody, and HBV total core antibody. We calculated the prevalence of immunity to each virus, and specifically targeted the difference for HBV immunity in those born after 1991, when implementation of universal HBV vaccination of newborns started in the US.

**Results:**

Between 09/01/2020 and 12/31/2023 we collected data on 1586 people living with HCV. The overall prevalence of HAV immunity was 46% (726/1586); for HBV, 36% (578/1586) were immune through vaccination (only HBV surface antibody positive), when divided by age group those born in 1991 and earlier had 34 % immunity compared to 52.5% for those born after 1991 (p< .0001). The prevalence of exposure to HBV was 24% (380/1586) with 7 having Hep B s Ag positive, and 126 only Hep B core antibody positive; when divided by the same age group categories, only 7 % of those < 32 years old had HBV exposure, as compared to 26% of those 33 years and older at a p value < .0001. Prevalence of people with all 3 markers negative for HBV (requiring vaccination) is 40% (628/1586); with no statistical difference between the two-age group, 40.5% for < 32 years old, and 39.5% for those 33 years and older.

**Conclusion:**

Our study shows an urgent need to implement hepatitis A and hepatitis B vaccinations in people living with HCV in NJ, almost half of them are not immune to either virus. On the other hand, it also highlights the success of universal vaccination of newborn for HBV, since we found a statistically significant number of those born after 1991 to be immune and have less HBV exposure.

**Disclosures:**

Jihad Slim, MD, FACP, AbbVie: Grant/Research Support|AbbVie: Honoraria|AbbVie: Speaker Bureau|Gilead Sciences, Inc.: Grant/Research Support|Gilead Sciences, Inc.: Honoraria|Gilead Sciences, Inc.: Speaker Bureau|Merck: Grant/Research Support|Merck: Honoraria|Merck: Speaker Bureau|Theratechnologies: Advisor/Consultant|Theratechnologies: Honoraria|ViiV Healthcare: Advisor/Consultant|ViiV Healthcare: Grant/Research Support|ViiV Healthcare: Speaker Bureau

